# Altered tactile abnormalities in children with ASD during tactile processing and recognition revealed by dynamic EEG features

**DOI:** 10.3389/fpsyt.2025.1611438

**Published:** 2025-09-16

**Authors:** Wenjie Wang, Yuan Liu, Ping Shi, Jiayu Zhang, Guoyao Wang, Yuanyuan Li, Wei Liu, Dong Ming

**Affiliations:** ^1^ Academy of Medical Engineering and Translational Medicine, Tianjin University, Tianjin, China; ^2^ Haihe Laboratory of Brain-Computer Interaction and Human-Machine Integration, Tianjin, China; ^3^ Department of Psychology, Tianjin Children’s Hospital, Tianjin, China; ^4^ Tianjin Children’s Hospital, Tianjin, China; ^5^ Tianjin University Children’s Hospital (Tianjin Autism Children’s Rehabilitation Technology Resource Center), Tianjin, China

**Keywords:** ASD, tactile processing, ERP, electrotactile, EEG

## Abstract

**Introduction:**

Autism Spectrum Disorder (ASD) is a neurodevelopmental disorder characterized by sensory processing abnormalities, particularly in tactile perception, highlighting the need for objective screening methods beyond current subjective behavioral assessments.

**Methods:**

This study developed a portable electro-tactile stimulation system with EEG to evaluate tactile processing differences in children with ASD (n=36) versus typically developing controls (n=36).

**Results:**

Revealing significantly reduced ERP amplitudes at key processing stages: P200 at FP2 (F(1,70)=10.82, p=0.0454), N200 at F3 (F(1,70)=58.33, p<0.0001), and P300 at C4 (F(1,70)=45.62, p<0.0001). Topographic analysis identified pronounced group differences (>10ìV) across frontal, central, and parietal regions (F8, FC5/6, CP1/2/5/6, Pz, Oz), with ASD children exhibiting prolonged but less efficient tactile discrimination and compensatory prefrontal activation (FP2 CV: p=0.043). The paradigm demonstrated strong reliability (CV ICC: ASD=0.779, TD=0.729) and achieved 85.2% classification accuracy (AUC=0.91) using ANN, with optimal performance from F8 P300 features (sensitivity=87.5%, specificity=83.7%).

**Discussion:**

These findings provide an objective, efficient (15-minute) screening method that advances understanding of tactile processing abnormalities in ASD and supports the development of physiological biomarkers for early identification, overcoming limitations of questionnaire-based approaches.

## 1 Introduction

ASD is identified as an early-onset neurodevelopmental disorder, the etiology of which poses a significant challenge on a global scale (1). Despite ongoing research efforts, the specific causes behind ASD remain largely undetermined, paralleled by a steady increase in diagnostic rates annually (2). Evidence supports early intervention as a pivotal strategy in ameliorating and alleviating the symptomatic expressions associated with ASD (3), underscoring the essence of precocious diagnosis or screening initiatives (4). Studies have shown that ASD symptoms typically manifest in early childhood, with 75-88% of children with ASD exhibiting signs within the first two years of life. The earlier the intervention, the more effective the treatment; research indicates that interventions before the age of three yield the most significant outcomes. However, in China, the median age for early ASD screening is 39 months, with a confirmed diagnosis typically occurring approximately one year after initial screening (5). Yet, empirical insights suggest that interventions administered prior to the completion of the third year of life yield superior outcomes (6), highlighting the critical need for the advancement of early screening timelines to effectively address ASD.

The decade subsequent to the DSM-5’s introduction has been characterized by a reiterated acknowledgment of perceptual abnormalities as a salient feature of ASD (7). Results from questionnaires administered to parents have shown that sensory perception (8), especially tactile sensitivity (9), serves as a typical marker of abnormalities. Moreover, some researchers believe that evidence from mouse models indicates that deficits in peripheral sensory neurons can contribute to ASD (10). Nevertheless, the assimilation of effective recognition and screening methodologies for these abnormalities remains insufficient within both clinical settings and the broader community context. An enhancement in awareness concerning these early markers is anticipated to facilitate the promotion of earlier screening and diagnosis endeavors, consequently enabling the provision of timely and efficacious interventions for individuals affected by ASD.

In recent years, some researchers have focused on quantifying tactile sensitivity issues in ASD using behavioral experiments or questionnaires. Tavassoli and colleagues (11) utilized a vibrotactile stimulation device to compare tactile sensitivity between individuals with ASD and TD participants. Participants were required to identify the stimulated finger after perceiving a weak vibration on either the index or middle finger. The results showed that higher tactile thresholds were significantly associated with more pronounced ASD traits. Anne and colleagues (12) developed a tactile frequency discrimination task using electrical stimulation. The ASD group demonstrated slower adaptability when adjusting to new stimulus frequency ranges, indicating that individuals with ASD differ from TD individuals in processing sensory inputs. This highlights the potential for incorporating tactile sensitivity into ASD auxiliary diagnostics. However, behavioral methods and questionnaires rely heavily on the cognitive abilities of participants. Consequently, these experiments are typically conducted on ASD individuals aged 18 and older, leaving a significant gap in the application of objective tactile sensitivity measures for early ASD screening, particularly for children under the age of 3.

Therefore, developing an evaluation paradigm that minimizes cognitive demands and does not require participants to perform tasks is critical for advancing research methodologies and improving early ASD detection. Electroencephalography (EEG) offers a promising approach in this regard. Recent studies have used EEG to investigate the neural specificity of individuals with ASD, primarily focusing on resting-state EEG (13, 14)and visual evoked potentials (VEP). Resting-state EEG assesses functional connectivity during rest, revealing atypical patterns in ASD, such as reduced low-frequency (delta, theta) connectivity and excessive high-frequency (beta, gamma) connectivity (13, 14), which correlate with ASD’s cognitive and behavioral traits. It is simple to conduct and suitable for all ages, including low-functioning individuals, but lacks task-related cognitive engagement, limiting its ecological validity. VEP (15), on the other hand, records brain responses to visual stimuli (e.g., faces, patterns) and highlights significant differences between ASD and TD individuals. ASD individuals often show atypical N170 waveforms in face processing tasks and abnormalities in P100 and P300 during visual pattern processing (15). While VEP effectively identifies perceptual and cognitive deficits, its reliance on visual stimuli and participant cooperation limits its applicability for individuals with visual impairments or attention deficits. Tactile paradigms bridge these limitations by requiring minimal cognitive effort while capturing task-related brain activity, offering a practical and objective alternative for studying cognitive processes in ASD. To date, research on tactile EEG in ASD remains extremely limited. The few existing studies predominantly focus on brain responses to simple tactile stimuli, often overlooking the assessment of tactile resolution. Notably, Piccardi et al.’s study employed a single-point tactile paradigm to investigate the neural markers of tactile sensory processing in 10-month-old infants at high risk for ASD or Attention Deficit Hyperactivity Disorder (ADHD). The neural response results revealed significantly reduced alpha wave desynchronization in high-risk ASD infants. These findings highlight the potential of tactile EEG paradigms for early identification of neural markers associated with ASD (16). However, the use of single-point, unvarying tactile stimuli does not allow for the investigation of tactile resolution, resulting in the largest tactile processing difference in ASD being overlooked. Thereby leaving a significant gap in the nuanced understanding of tactile processing and tactile resolution in ASD.

The current gaps in ASD tactile processing research are threefold: behavioral methods rely on cognitive abilities, limiting use in children under 3; EEG paradigms like resting-state EEG lack task engagement, and VEP depends on visual stimuli and cooperation; existing tactile EEG studies, such as Piccardi et al.’s, use simple stimuli, neglecting tactile resolution. Addressing these gaps requires developing objective, low-cognitive-demand tactile EEG paradigms capable of capturing multidimensional tactile processing characteristics, particularly for early ASD detection.

Previous research on the mechanisms of tactile event-related potential (ERP) has predominantly focused on typically developing individuals. Among the electrophysiological metrics, the ERP components associated with the brain’s processing of tactile stimuli include P100 (17, 18), N140 (19), P200 (20), N200 (21), and P300 (20) components are associated with the temporal processing of tactile stimuli in the brain. The P200 component, occurring during the mid-stage of tactile temporal processing, has been identified as a critical marker for distinguishing between multi-level tactile sensations. In contrast, the P300 component, observed during the late-stage of tactile temporal processing, exhibits a strong correlation with subjective judgments of tactile sensations (22). However, the electrophysiological performance of ASD in tactile temporal processing, especially in these components compared to TD, and the mechanisms of ASD in recognizing multi-level tactile stimuli, require further exploration.

The contribution of this study is its utilization of EEG to provide an objective assessment of tactile response resolution in individuals with ASD compared to TD counterparts, with the aim of elucidating distinct neurophysiological patterns and establishing tactile EEG as a robust physiological biomarker for ASD. In this study, we used a multi-channel tactile electrical stimulation and wireless EEG synchronization acquisition system suitable for ASD. We also developed a paradigm for multi-level pressure tactile stimuli. By sequentially analyzing ERP components, cortical activity, and the coefficient of variation (CV) in tactile information processing, we aimed to investigate cognitive deficits in ASD, particularly the dynamic information processing abnormalities during tactile recognition.

## 2 Materials and methods

### 2.1 EEG-based experimental system for assessing tactile resolution

**Figure 1:**
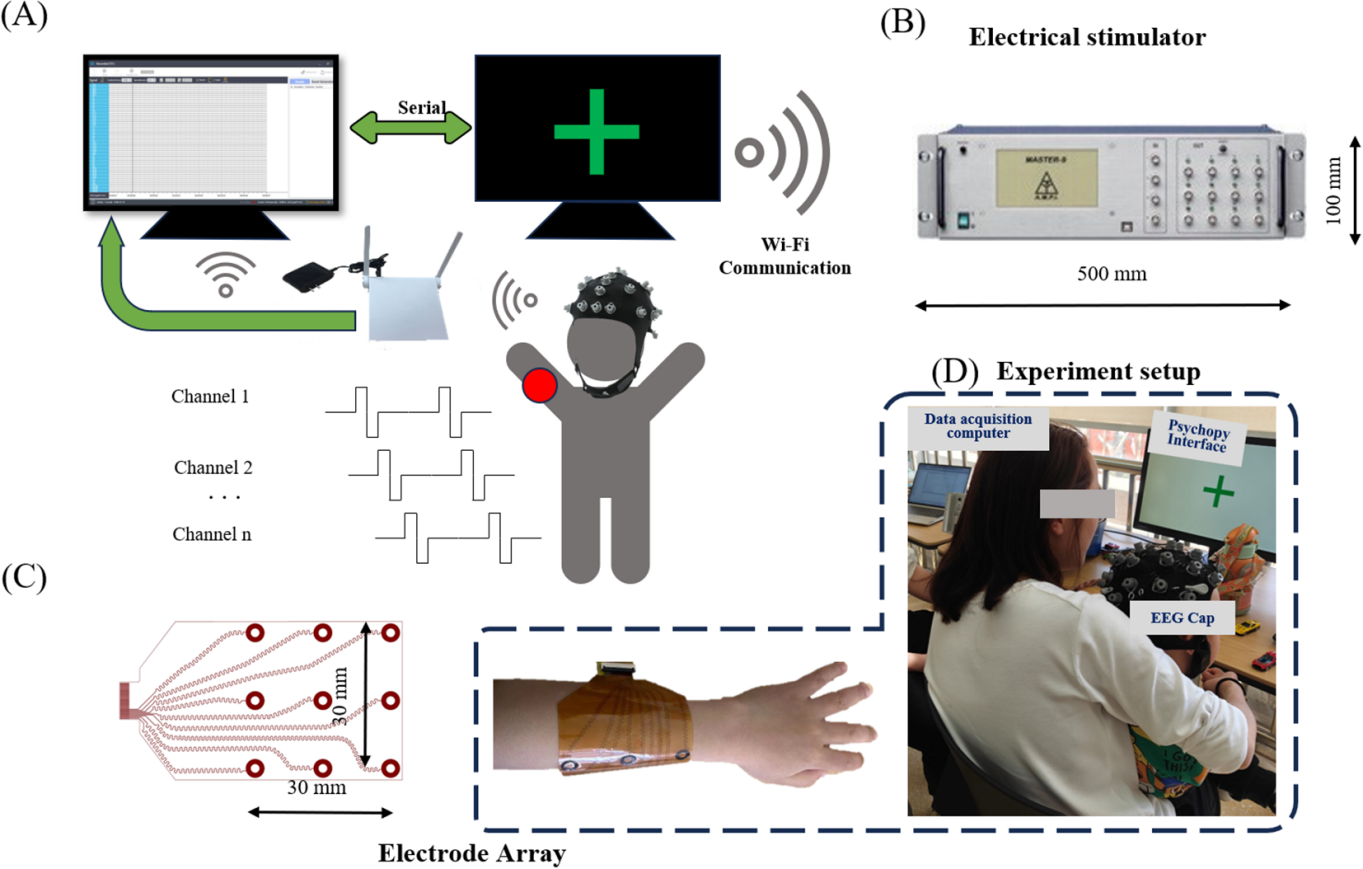
EEG-Based Experimental System for Assessing Tactile Resolution. The system consists of three main components: a Multimodal electro-tactile stimulator, a wireless EEG acquisition module, and array-style flexible electrodes. **(A)** Wireless EEG acquisition module **(B)** Multimodal electro-tactile stimulator. **(C)** Array-style flexible electrodes. **(D)** Experimental Setup.

In this paper, we present an integrated EEG-based system specifically designed for the assessment of tactile resolution (refer to **Figure 1**). The system consists of three main components: a multimodal electro-tactile stimulator, a wireless EEG acquisition module, and array-style flexible electrodes. The electrical stimulation system measures 500×100 mm (as illustrated in **Figure 1B**) and is portable, can be directly placed on a desktop. The array-style electrodes are flexible, ensuring they meet the requirements for portability. This compact size and flexibility allow the system to maintain effective electrical stimulation even if there are slight movements by the subject during the experiment.

#### 2.1.1 Multimodal electro-tactile stimulator

Electrical stimulation provides precise, controllable, and direct neural activation for tactile sensations, offering rapid response (brain responses can be observed immediately without delay from the moment of stimulation via EEG), scalability (electrical stimulation systems can be scaled to stimulate multiple sensory points simultaneously), technological integration (the system’s area can be reduced, making the entire system wearable), and minimal invasiveness (ASD do not experience discomfort during the experiment and does not pose a threat to their health), making it superior to other methods (vibration feedback, mechanical stimulation, pneumatic and hydraulic systems, thermal feedback, ultrasonic haptics, magnetic and chemical stimulation, optogenetics, and light-based feedback) in applications like neuroprosthetics and human-computer interaction (23). The stimulation was delivered to the median nerve in the left hand (22, 24). Electrical impulses are output by the electrical stimulator (Master-9, AMPI, Israel) and encoded via custom software based on MATLAB R2024b (MathWorks, MA, USA) with the toolbox Psychtoolbox. Commands for electrical stimulation, including current intensity, pulse width, and frequency, can be precisely controlled and monitored in real-time via PC through WiFi.

#### 2.1.2 Array-style flexible electrodes

In this study, electrodes need to be placed in contact with the human epidermis to input the stimulation current. Flexible printed circuit electrodes were used, as shown in **Figure 1D**. Based on our previous threshold experiments with normal subjects, the FPC electrodes adopted a ring-shaped unipolar configuration (25) (outer diameter: 8mm, inner diameter: 4mm) and directly input pulse modulation signals. The axial distance between the electrodes was set to 30 mm, and the circumferential distance was set to 30 mm. To increase the contact area between the electrodes and the skin and prevent burns caused by excessive local current due to uneven contact, conductive gel was used as the contact medium between the electrode and the skin.

#### 2.1.3 Wireless EEG acquisition module

Given the difficulty ASD patients experience in maintaining a stable seated posture for prolonged periods, often displaying involuntary head movements or body shifts, this study employed the NeuSen W wireless EEG amplifier system (Neuracle). The amplifier was positioned at the back of the head, tightly adhering to the scalp, and directly connected to the electrode cap to mitigate the effects of electrode cap cable drift caused by minor head and body movements. This setup minimized disruptions to the acquisition of raw EEG data (26). To streamline the experimental setup, a saline-based electrode cap was selected, allowing for immediate data collection upon placement on the participant. The system includes 32 scalp electrodes configured according to the international 10/20 system, with the reference and ground electrodes positioned at CPz and the forehead, respectively. EEG signals were recorded using a bandpass filter, with a sampling rate of 1000 Hz and a frequency range of 0.5 to 100 Hz (27).

During tactile stimulation for the subjects, the stimulus computer dispatches a sequence embedded with electrical stimulation parameters to the stimulation system, concurrently transmitting time stamps to the EEG acquisition computer through a serial port. Subsequent to EEG signal collection, the saline electrode cap transmits these signals to the EEG acquisition computer via WiFi.

### 2.2 Experimental paradigm

#### 2.2.1 Participants

A total of 72 participants were enrolled in the experimental study. 36 subjects diagnosed with Autism Spectrum Disorder (ASD), aged between 1 and 5 years (mean age = 3 years, range: 1 year 2 months to 5 years 2 month), were recruited from the Psychological Outpatient Clinic and Autism Intervention Center at the Children’s Hospital affiliated with Tianjin University. The inclusion criteria for the ASD group were as follows: For children under 3 years old, early screening was conducted using the Autism Behavior Checklist (ABC), a widely validated tool for identifying ASD characteristics in young children. The ABC scores were combined with clinical observations by experienced pediatricians to identify children highly suspected of having ASD. While these methods are not equivalent to formal diagnoses using standardized tools such as ADOS or ADI-R, they are appropriate for early identification and screening in this age group. To ensure consistency within the group, the same ABC scale was also used for children over 3 years old (28), All children with ASD aged 3 and above were initially screened using the ABC scale and clinically diagnosed by two senior physicians based on DSM-5 criteria, with 85% of cases confirmed by ADOS-2 assessment. Exclusion criteria included the presence of known genetic conditions (e.g., tuberous sclerosis), significant head trauma, neurological disorders or history thereof (e.g., epilepsy), severe physical illness, metallic implants in the head or neck, or current use of psychotropic medications. A control group of 36 age-matched typically developing (TD) children was recruited from the Child Health Department of the same hospital through public postings. None of the control participants had been suspected by pediatricians of having ASD or any other developmental disorders. The study protocol was reviewed and approved by the Ethics Committee of the Children’s Hospital affiliated with Tianjin University (2023-IITKY-005). Informed consent was obtained from the guardians of all participants prior to the commencement of the study. Guardians were informed that participation was voluntary and that they could withdraw their child from the study at any point without any consequences.

#### 2.2.2 Electrical stimulation tactile paradigm

The type and intensity of tactile sensations elicited by electrical stimulation are contingent upon various parameters of the stimulus. Specifically, the frequency of electrical stimulation correlates with the type of tactile sensation, with 100 Hz linked to the perception of pressure (29). Moreover, the pulse width and amplitude of the stimulation influence the tactile intensity. Utilizing our prior up-down threshold experiments conducted on healthy adults (22), we established that at a fixed frequency of 100 Hz and an amplitude of 2 mA, adjusting the pulse width provides a normal discriminative resolution of five levels of pressure sensation. These levels, corresponding to incremental tactile sensations from low to high, are defined by pulse widths of 20, 100, 200, 300, and 600 microseconds. In this study, we focused on the commonly perceived sensation of pressure (25), adopting the widely used pressure-induction paradigm in the field of electro-tactile stimulation. We selected the tactile benchmarks from our previous experiments (22) as the standard stimulus paradigm with five pressure levels (L1/L2/L3/L4/L5), corresponding to pulse widths of 20, 100, 200, 300, and 600 microseconds, respectively.

#### 2.2.3 Experimental procedure

**Figure 2:**
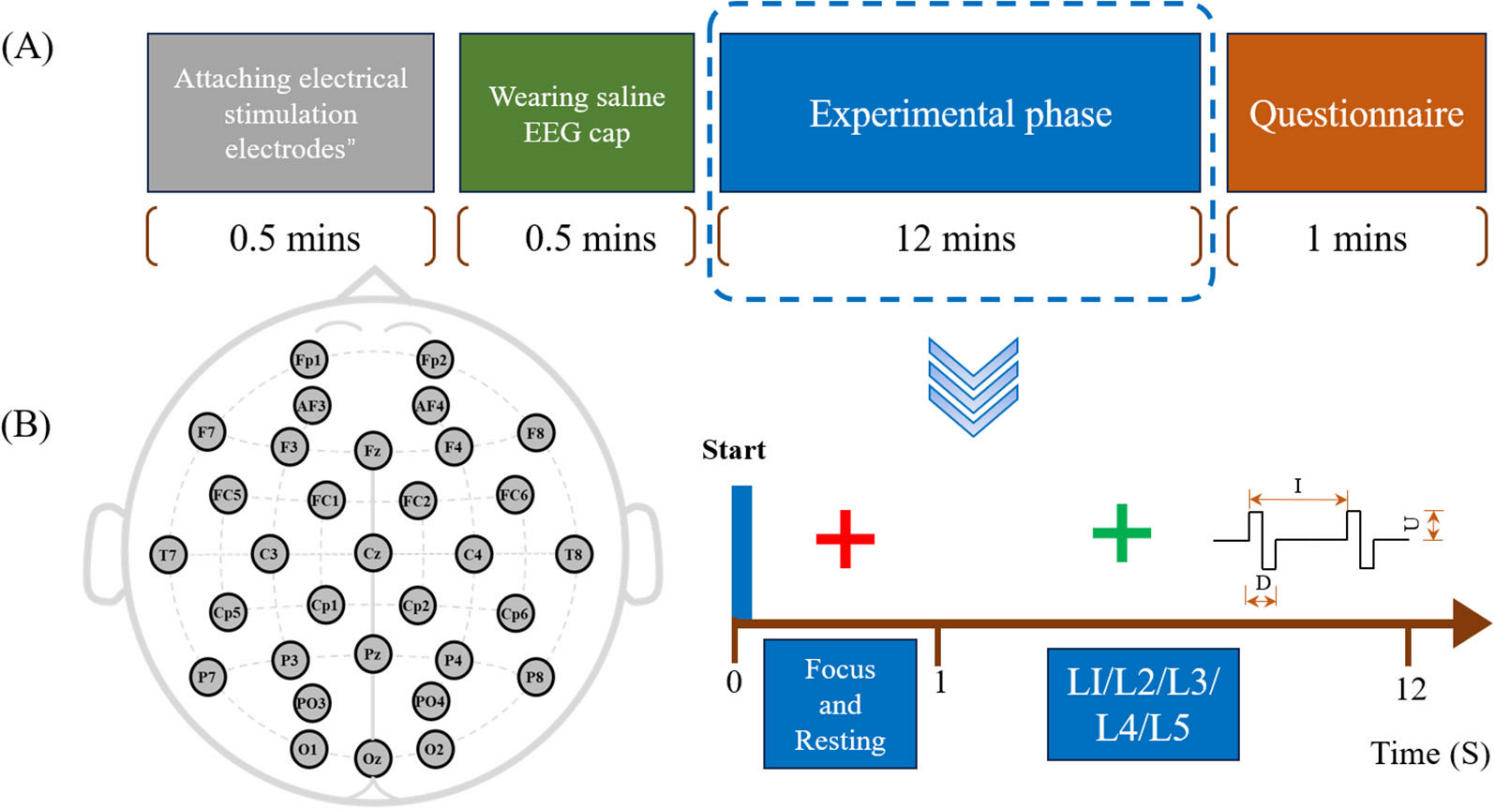
**(A)** Experimental procedure: Colored blocks represent different steps, with the total procedure lasting 15 minutes, including a 12-minute experimental session. **(B)** EEG acquisition process: Includes 1-minute resting-state recording and five levels of random tactile stimulation.

The entire experimental procedure lasts 15 minutes, as shown in **Figure 2A**. Subjects are required to sit in front of a computer screen and cooperate with the experimenter to attach the electrodes and wear a saline EEG cap (for subjects S10 and S12, who are infants under the age of 2, the preparation and experimental procedure are completed with the assistance of their guardians). We conducted all experiments in a sound- and electromagnetically-shielded chamber with rigorously controlled environmental conditions (23 ± 1°C, <30 dB background noise) using carefully screened participants with no prior electrotactile experience. The EEG acquisition process is illustrated in **Figure 2B**. The experiment begins with the collection of resting-state EEG for one minute. Stimulation starts when a green cross appears on the screen. Five levels of stimulation are presented randomly, with each level repeated 80 times. Each stimulation lasts for 1 second, followed by a 0.5-second rest period. At the end of the experiment, participants are asked two questions: 1. Please describe the sensation you just experienced. 2. Was the stimulus you just felt painful or itchy?

After administering the questionnaire, we documented each participant’s verbal description of their tactile experiences whenever such descriptions were provided. Drawing upon our previous research with typically developing adults, we determined that a correct response should describe the tactile stimulus as a sensation of pressure, free from pain or discomfort.

### 2.3 Data preprocessing

#### 2.3.1 EEG data preprocessing

The analysis was conducted using custom MATLAB scripts. Initially, artifacts such as blinks and teeth clenching were removed (5 conditions * 80 trials * 36 participants = 14400 trials, with 217 trials removed), and bad channels were identified using the channel power spectrum before being interpolated (A total of 72 participants with 32 channels each, averaging 0.81 channels per participant requiring interpolation). Next, detection task data were segmented into 1500 ms epochs, ranging from -500 to 1000 ms relative to stimulus onset, with baseline correction applied to eliminate drift. A third-order Butterworth bandpass filter (0.01–12 Hz) was then utilized to remove electrical stimulation artifacts and other noise. Next, blinking artifacts, horizontal eye movements, vertical eye movements, and other generic discontinuities were removed using Independent Component Analysis (ICA) through the ADJUST plugin within the EEGLAB toolbox in MATLAB (30). Finally, ERP waveforms and brain topographic maps were generated. Separate analyses were performed for the lateral recording sites C3/4, P3/4, as well as the prefrontal lobe FP1/2, and the frontal electrode F3/4 (31), in previous tactile ERP studies, these electrodes were found to be associated with tactile discrimination tasks (24). For each task condition (i.e., L1/L2/L3/L4/L5), all artifact-free trials were extracted and trial-averaged for each participant. Subsequently, in the [50:100], [100:150], [150:200], [250:300], and [300:350] ms time windows, the mean amplitudes within the 20-ms time window centered at the peak were defined as the amplitudes of P100, N140, P200, N200, and P300, respectively, particularly in the regions generating these components. To investigate the ERP differences between TD and ASD under these five conditions, two-way analysis of variance (ANOVA) and *post hoc* tests were used (with the independent variables being Group (TD and ASD) and Condition (the five different experimental conditions), and the dependent variable being ERP amplitude).

#### 2.3.2 Analysis of coefficient of variation

The coefficient of variation (CV) serves as a quantitative measure of neural signal variability that has been extensively employed in electrophysiological research (32). This metric demonstrates particular utility in assessing information processing efficiency across various cognitive domains, including linguistic processing and the evaluation of cognitive impairment. From a statistical perspective, reduced CV values reflect a more peaked distribution (leptokurtic), while elevated CV values indicate a flatter distribution (platykurtic) (33). Stable brain activity results in smooth coefficient changes, whereas stimulus-induced changes in EEG signal amplitude cause sharp coefficient changes (32, 33). By comparing the CV values of EEG signals across different time periods, O’Reilly et al. can evaluate the oscillatory response variability of the brain to external stimuli to assess changes in brain maturity in infants (34). Segning et al. applied pain stimulation using external capsaicin and found that the CV of EEG signals differed between patients and healthy individuals under stimulation. These results support the use of CV of EEG signals as a quantitative measure to objectively identify the presence of chronic fibromyalgia (35). In our previous research, we observed that different healthy adults demonstrated consistent dynamic CV values during multi-level tactile processing. Notably, significant increases in CV values were detected at 100 ms, 200 ms, and 300 ms post-stimulus onset, whereas the CV values at other time intervals remained nearly negligible (22). To further describe sensation processing efficiency over time within subjects, we computed the CV for each time segment waveform across five pressure levels. The ERP waveform, consisting of five components (0–400 ms post-stimulation) for each pressure stimulus, was selected for analysis. The ERP waveform was divided into segments using a sliding window approach (10 ms window width, 1 ms step length). For each segment, the CV was calculated to reflect tactile processing efficiency at each time point, defined as CV= S.D./MEAN. The calculated values represent the standard deviation and mean of ERP amplitudes at each point within the time window.

#### 2.3.3 Reliability assessment

To ensure the reliability of EEG data from ASD and TD participants, we calculated the intraclass correlation coefficient (ICC) (36) for each participant’s EEG features (e.g., ERPs and CV values) using custom MATLAB scripts. ICC was computed using a two-way random-effects ANOVA model to assess absolute agreement across measurement conditions (37). The formula for ICC is:


$$icc=\frac{MS_{R}-MS_{E}}{MS_{R}+(k-1)\cdot MS_{E}+\frac{k}{n}(MS_{C}-MS_{E})}$$

where represent mean squares between subjects, conditions, and error, respectively; k is the number of conditions, and n is the number of participants. ICC values > 0.75 indicate excellent reliability, while values < 0.5 suggest poor reliability. Participants with low ICC values were excluded to ensure data quality.

#### 2.3.4 Classification of ASD and TD groups

The machine learning analysis employed three distinct classifiers - Support Vector Machine (SVM) with radial basis function kernel, Linear Discriminant Analysis (LDA), and a three-layer Artificial Neural Network (ANN) - to differentiate ASD from TD participants. We performed 10-fold stratified cross-validation to ensure robust evaluation, maintaining equivalent class distribution (50% ASD *vs* 50% TD) across all training/testing splits. The models utilized identical input feature sets comprising: (1) peak amplitudes of P100, N140,P200, N200, and P300 components from frontal and central electrodes, and (2) coefficient of variation (CV) values calculated across 200-400ms post-stimulus windows.

## 3 Results

### 3.1 Subjective question results

In the subjective Q&A section, for the first question, in the ASD group, 2 participants (S10, S12) were too young to describe the sensation, 4 participants (S2: “It felt like a mosquito bite,” S13: “It felt like an electric current,” S20 and S31: “It felt like a vibration”) were able to fully describe the sensation, 5 participants (S1, S5, S21, S24, S32) responded with screams, and the remaining 25 participants were unable to describe the sensation due to language development issues. In the TD group, similarly, 2 participants (S13, S14) were too young to describe the sensation, 12 participants described the sensation similarly to typically developing adults (such as the pressure of a press), 1 participant (S17) described it as an electric current, and the remaining 21 participants mostly described the sensation as a vibration. For the second question, neither the ASD group nor the TD group reported the tactile electrical stimulation as painful or itchy.

During data analysis, a score of 1 was assigned to each correct description, whereas incorrect or missing descriptions were assigned a score of 0. For example, in the ASD group, responses were coded as 0, 0, 0, 0, 0, 0, 0, 0, 0, 0, 1, 1, 0, 0, 0, 0, 0, 0, 0, 0, 0, 0, 0, 1. Subsequent statistical analysis indicated that there was no significant difference in questionnaire scores between the ASD and TD groups.

### 3.2 ERP results

**Figure 3:**
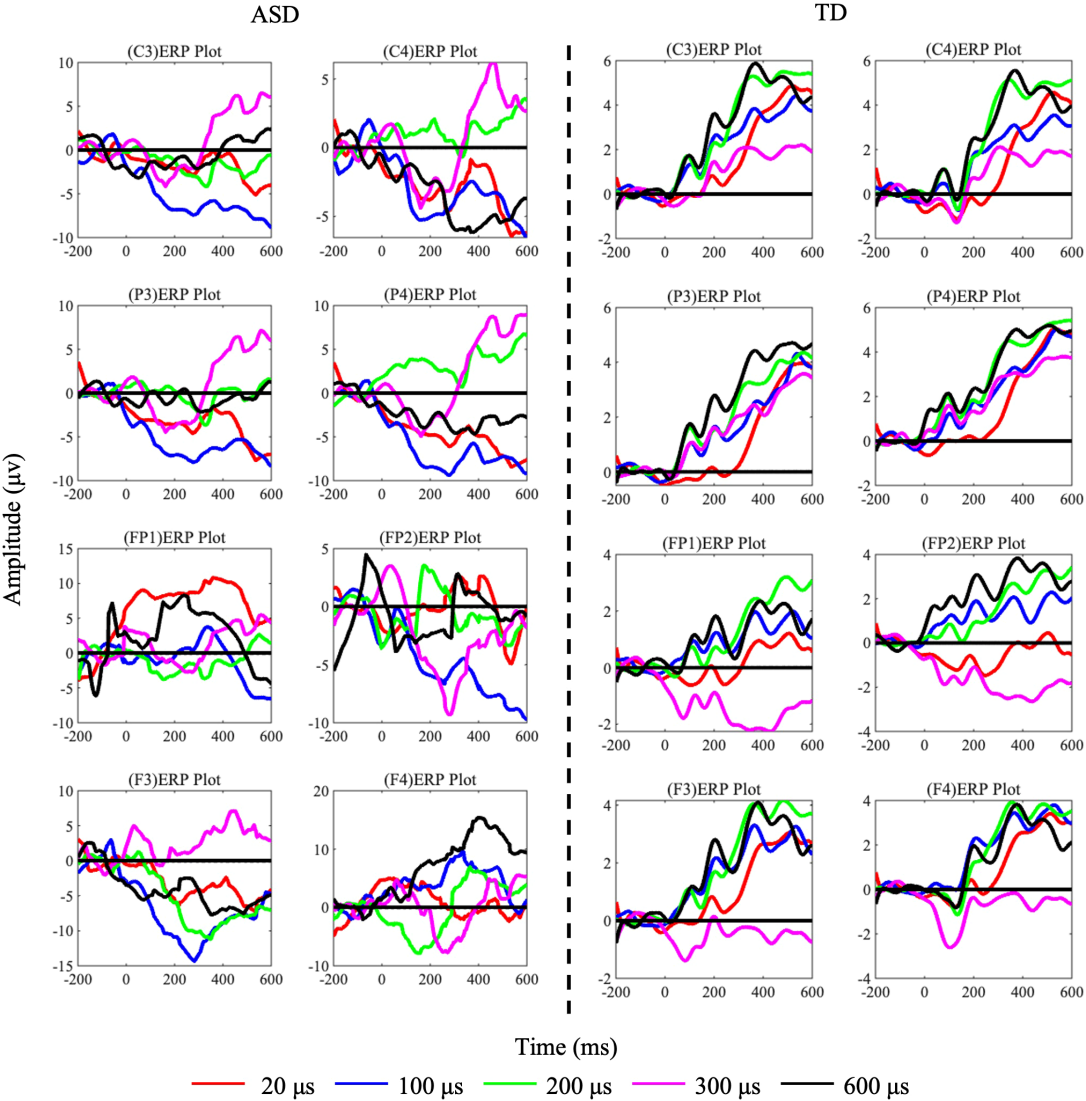
ERP components of TD and ASD under each task condition (L1/L2/L3/L4/L5) with pulse widths of 20 us (red), 100 us (blue), 200 us (green), 300 us (pink), and 600 us (black). The left side shows data from 36 ASD subjects, and the right side shows data from TD subjects.

The grand-averaged EEG response exhibited the anticipated somatosensory evoked potential components. The mean ERP waveforms from these eight electrodes are depicted in **Figure 3**. The statistical analysis employed a two-factor analysis of covariance (ANCOVA) model to examine five components (P100, N140, P200, N200, and P300) amplitude differences while controlling for age effects. The model incorporated diagnostic group (ASD *vs*. TD) as a between-subjects factor, tactile stimulus level (5 ordered levels) as a within-subjects factor, and participant age as a continuous covariate, Using MATLAB’s fitlm function with Type III sums of squares, we specified the full factorial model including the group × stimulus interaction term to test whether group differences varied across stimulus intensities. Age was included as a covariate to account for potential developmental influences on neural responses. *Post-hoc* (LSD) analyses of age-adjusted marginal means were conducted using multcompare, with categorical variables properly specified in the model. Effect sizes were calculated as partial eta-squared (η²p) for significant effects, and all analyses employed an alpha level of 0.05 for statistical significance testing. The F3 N200 component showed remarkable Group differences (F(1,70)=58.33, p<0.0001, η²=0.834), with age explaining 8.7% of variance (β_age=-0.29, p=0.015). Notably at C4: (1) The N200 Group effect became more pronounced (F(1,70)=72.15, p<0.0001, η²=0.892) with minimal age influence (Δη²=0.008); and (2) For P300, Group differences strengthened (F(1,70)=45.62, p<0.0001, η²=0.854). No significant Group×Task interactions emerged (ps>0.1). Since our ultimate goal was to distinguish between ASD and TD, we conducted a *post hoc* power analysis for between-group differences (ASD *vs*. TD) to ensure sufficient statistical power. The results for the significant electrodes and components all yielded power values >0.8. For the N200 component at electrode F3: *Post hoc* power analysis for the group effect (ASD *vs*. TD) revealed a power > 0.99 (Cohen’s f = 1.71, α = 0.05, n = 36 per group), indicating sufficient sensitivity to detect large effects. A sample size of 9 per group provides 80% power to detect effects with f ≥ 0.82. A significant group difference was observed in N200 amplitude, with ASD showing a 10 μV increase compared to TD [95% CI: 4.2, 15.8].

### 3.3 Cortical activity

Through ERP analysis, temporal domain differences between ASD and TD groups were identified. Next, spatial domain differences were analyzed using brain topography maps. (1) Calculating the brain region differences between the ASD and TD groups when receiving tactile stimulation: The ERP signals for five tactile stimulation conditions were averaged across three components and mapped onto brain topography maps, calculating the amplitude differences between ASD and TD groups across 30 electrodes. (2) Calculating the brain region differences between the ASD and TD groups during the tactile discrimination task for the five stimuli: The temporal data were averaged to compute the amplitudes of the five levels. To further examine the differences between the two groups in the tactile discrimination task, a one-way ANOVA was performed on the brain topography maps for the five tactile stimulation levels (factors: L1/L2/L3/L4/L5), and an LSD *post-hoc* test was applied. The p-values were mapped onto the brain topography maps.

**Figure 4:**
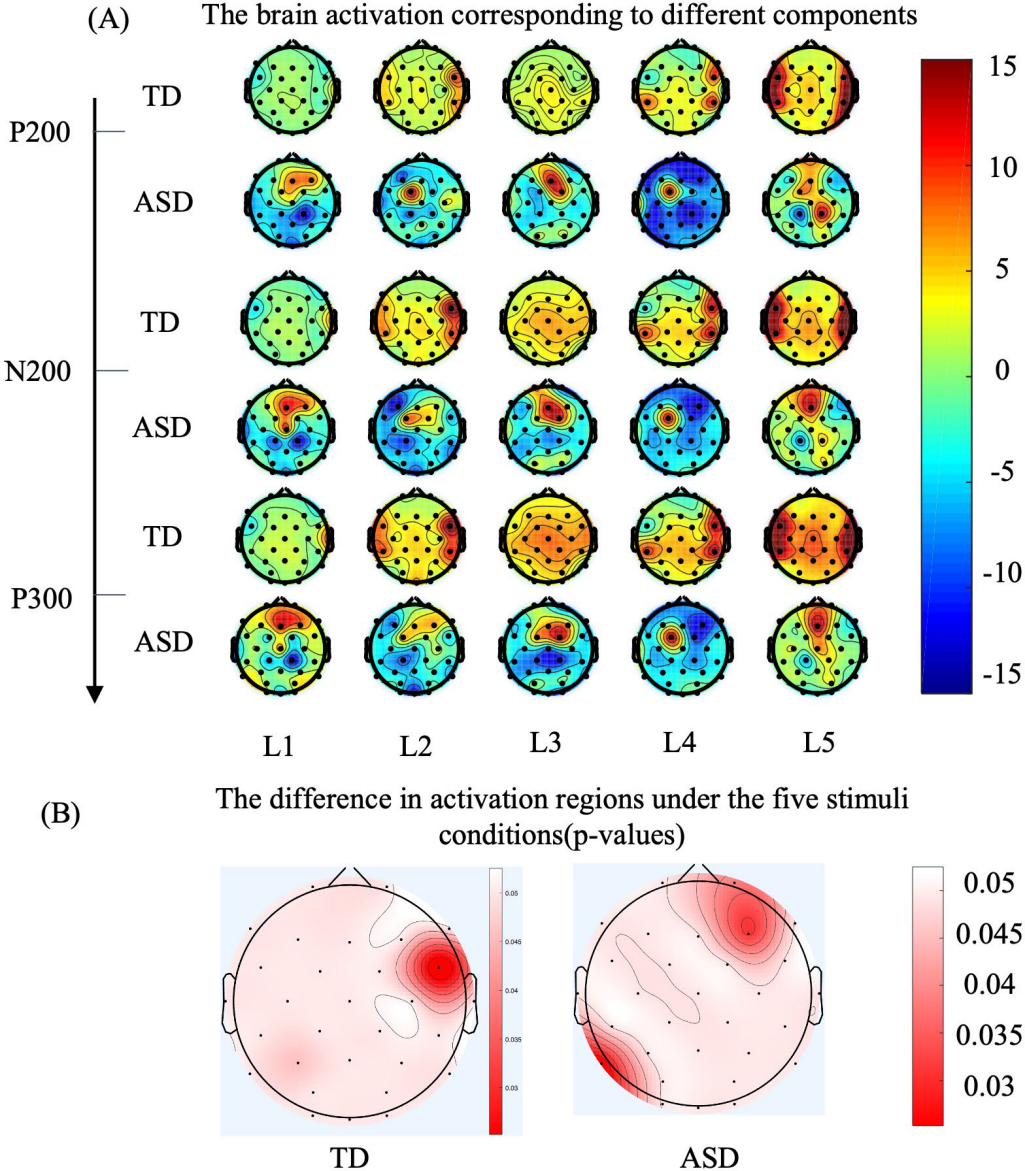
As for brain grand-averaged topographies, deeper red color represents higher evoked response, while deeper blue color represents lower evoked response. **(A)** The topographical distribution for the TD group is presented in the upper row, while the data for the ASD group are illustrated in the lower row. The three sets on the vertical axis represent the topographic maps corresponding to P200, N300, and P300, while the horizontal axis represents the five levels of tactile stimuli. **(B)** ERP differences (p-values) across five levels of tactile stimulation for 36 typically developing (TD) participants on the left brain topography map, and for 36 Autism Spectrum Disorder (ASD) participants on the right brain topography map. The color intensity indicates the magnitude of the differences, with redder areas representing larger differences (Factor: five levels of stimulation, Sample: 36 participants per group).

**Figure 4A** shows the brain topography maps of the ASD and TD groups, with data from 30 electrodes plotted on the brain topography. The ERP signals for three specific components were averaged. The red areas in the figure indicate stronger activation. As shown in **Figures 3** and **4**, the regions of increased and decreased activation are consistent with the scalp ERP components. The electrodes with the largest differences (greater than 10 μV) between the ASD and TD groups are: F8, FC5, FC6, T8, CP1, CP2, CP5, CP6, Pz, and Oz. To further observe the different performances of the two groups in the tactile discrimination tasks, We depicted the areas where TD and ASD children exhibited significantly different cortical activities during the five levels of tactile tasks. Specifically, as shown in **Figure 4B**, during tactile processing, TD children showed activation in the FC6 (p= 0.047 < 0.05, η^2^ = 0.45) and P3 (p= 0.042 < 0.05, η^2^ = 0.36), indicating significant differences in activation in response to the five levels of tactile stimulation. In contrast, ASD children showed significant differences in activation in the FP2 (p= 0.0319 < 0.05, η^2^ = 0.23), F7 (p= 0.0386 < 0.05, η^2^ = 0.235), and P7 (p= 0.024 < 0.05, η^2^ = 0.13) in response to the five levels of tactile stimulation.

### 3.4 Coefficient of variation

**Figure 5:**
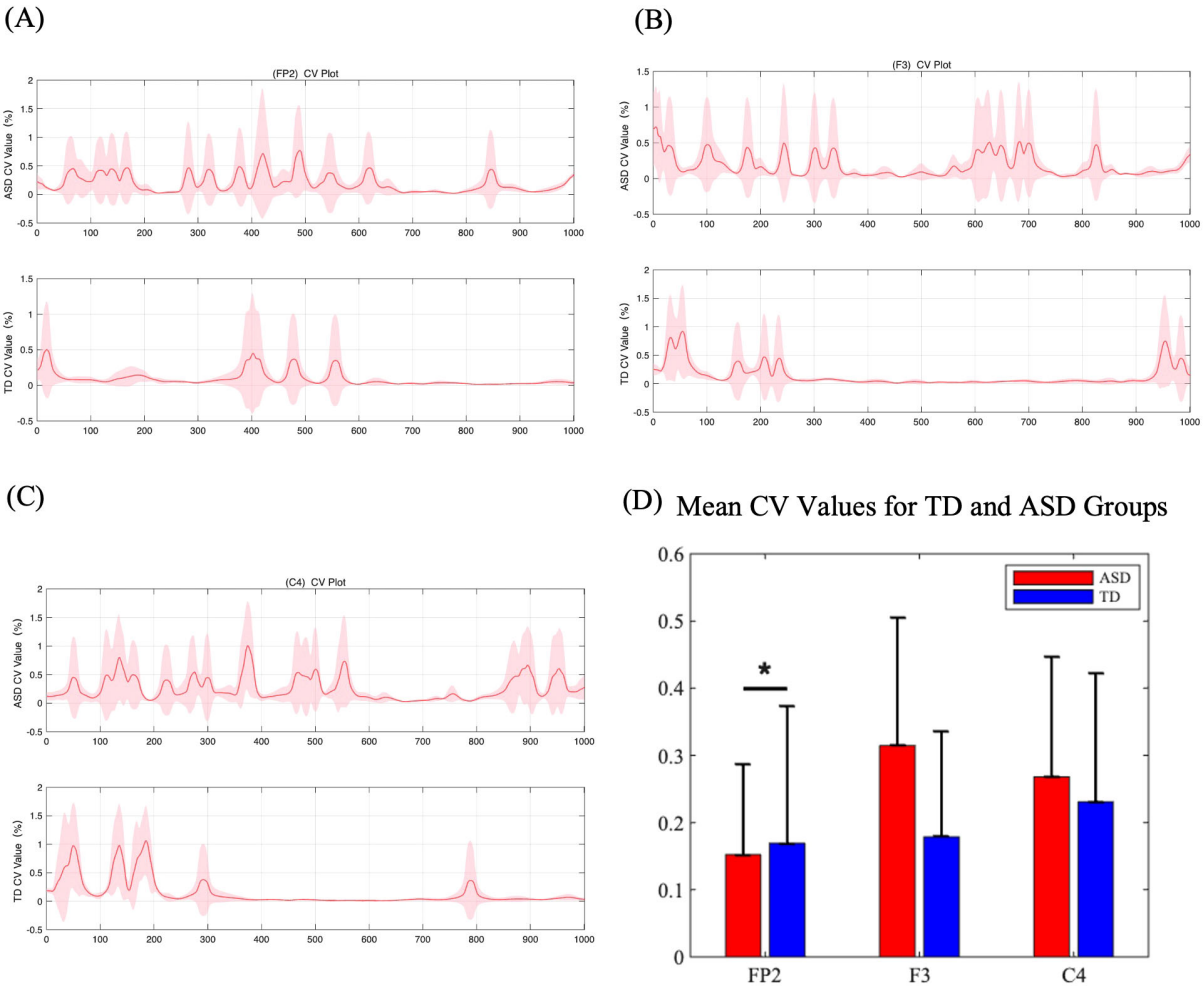
**(A-C)** (Color online) Coefficient of variation values for ERP amplitude between five grades of pressure. The curve represents the average CV values of the five grades, and the color blocks represent the standard deviations of the five grades. **(D)** Mean CV values for TD and ASD groups at three electrodes (FP2, F3, and C4). The bar plots show the mean CV values with standard error bars for each group.

Based on the three electrodes showing significant differences, signals from electrodes FP2, F3, and C4 were analyzed. As shown in **Figure 5**, compared to the TD group, the CV waveform of the ASD group is more dispersed throughout the entire time period. This suggests that the ASD brain is processing tactile information continuously over the entire duration, whereas the TD group concentrates tactile processing in the early phase of stimulus onset (0-400ms), with relatively flatter responses in other periods. This pattern can be observed in the FP2, F3, and C4 electrodes. **Figure 5D** presents the total CV values calculated over the entire time period. We performed a statistical analysis of the CV values for 36 ASD and TD participants using paired t-tests with FDR correction. The results revealed a significant difference at FC2, with ASD values significantly higher than those of TD (p= 0.043 < 0.05), indicating more intense information processing in the FC2 for ASD (22).

### 3.5 Test-retest reliability of EEG features in tactile tasks

The test-retest reliability of EEG features during tactile tasks was evaluated using intraclass correlation coefficients (ICC) for both ASD and TD children, the ICC values for ERP components related to different pressure levels and the coefficient of variation (CV) values were calculated. As shown in **Table 1**. Overall, TD children exhibited higher ICC values compared to ASD children, indicating greater consistency in their neural responses. For instance, the ICC values for ERP at Pressure Level 1 were 0.356 (poor) in ASD children and 0.568 (moderate) in TD children. Similarly, the CV values for pressure levels showed moderate to good reliability in both groups (ASD: 0.779, good; TD: 0.729, moderate). These results suggest that TD children demonstrate more stable neural responses across tactile tasks, while ASD children show lower consistency in ERP components associated with specific pressure levels. However, overall, the EEG data during tactile tasks demonstrated acceptable reliability, particularly for CV values and ERP components at higher pressure levels.

**Table 1:** Classification Performance of Different ERP Features in Discrimination.

Characteristics of classification	Rate of accuracy		
	SVM	LDA	ANN
ERP:P1	0.562 (FP1, P100)	0.623 (P3, N140)	0.712 (F8, P200)
ERP:P2	0.580 (FP2, P100)	0.554 (FP2, N140)	**0.847** (F8, P300)
ERP:P3	0.649 (CP6, P100)	0.558 (P4, P100)	0.840 (F8, P200)
ERP:P4	0.704 (P3, P100)	0.566 (FC1, P100)	**0.852** (FZ, P200)
ERP:P5	0.714(F7, P100)	0.608(FP1, P100)	0.840 (F8, P300)
CV	0.743 (FP1)	0.589 (FP1)	0.804 (T8)

### 3.6 Classification accuracy of EEG features

The classification accuracy of different EEG features under the static level recognition paradigm was evaluated using three machine learning algorithms: Support Vector Machine (SVM), Linear Discriminant Analysis (LDA), and Artificial Neural Network (ANN). As shown in **Table 1**, the highest classification accuracy was consistently achieved by ANN across all ERP features and CV values. For instance, for ERP at Pressure Level 1, ANN achieved an accuracy of 0.712 (F8, P200), while SVM and LDA achieved 0.562 (FP1, P100) and 0.623 (P3, N140), respectively. Similarly, for CV values, ANN achieved an accuracy of 0.804 (T8), outperforming SVM (0.743, FP1) and LDA (0.589, FP1). These results demonstrate that ANN is the most effective algorithm for classifying EEG features in the static level recognition paradigm, particularly for ERP components associated with higher pressure levels. The highest classification accuracy was obtained using an ANN classifier with F8 P300 features: The classification model achieved an overall accuracy of 85.2% with balanced performance across sensitivity (87.5%) and specificity (83.7%), yielding an estimated AUC of 0.91. While slightly below the optimal results mentioned previously, these metrics still demonstrate clinically meaningful discriminative power for ASD screening applications, as all values remain above the 80% threshold considered acceptable for preliminary diagnostic tools.

## 4 Discussion

Autism’s social deficits are often associated with sensory abnormalities. Most previous studies have utilized visual and auditory stimuli as physiological biomarkers. However, increasing research also recognizes that abnormal tactile sensations are a significant reason why individuals with Autism Spectrum Disorder find it challenging to engage socially. Although some clinicians have suggested incorporating tactile assessments into clinical scales, implementing such measures remains difficult due to the lack of objectivity and reliability of questionnaires, particularly because most patients are children with developmental delays and mild cognitive impairments. Clinicians require a general benchmark of tactile response to clinically diagnose the extent of tactile abnormalities.

According to the results of our subjective survey, even typically developing children with normal intelligence have difficulty fully describing tactile sensations, let alone children with Autism Spectrum Disorder. Furthermore, there were no significant differences between the two groups in the questionnaire results. Therefore, it is challenging to objectively differentiate the tactile perceptions of children with Autism Spectrum Disorder and typically developing children based on questionnaires alone, making it particularly important to find an objective assessment method for early identification.

Previous studies have used methods such as MRI to objectively assess tactile neural processing in ASD. The study revealed that, compared to TD participants, individuals with ASD exhibited a typical modulation of connectivity between the sensorimotor regions and the prefrontal cortex during tactile stimulation (38). However, research on how the brain dynamically allocates resources to accomplish tactile discrimination tasks is still scarce, especially studies that objectively assess the abnormal tactile resolution in ASD children. In this study, we used a portable electro-tactile stimulation system that can be applied to ASD children and utilized EEG to objectively describe tactile processing in ASD and TD children. Through a series of EEG analyses and statistical comparisons, we aim to reveal the mechanisms of tactile processing deficits in ASD children.

Generally, normal adults process tactile sensations and form motor decisions through three stages (22, 25): the early stage (P100, N140) involves the right somatosensory association cortex distinguishing tactile types; the middle stage (P200) involves the right primary somatosensory cortex recognizing different degrees of tactile stimuli and forming tactile characteristics; the late stage (N200 and P300) involves information reaching the prefrontal cortex, where different objects are recognized based on individual cognition.

According to the performance shown in **Figure 3**, TD children’s tactile processing can evoke typical tactile discrimination-related ERP components: P100–N140–P200–N200–P300. However, the early components in ASD children are not as typical as those in TD, and only P200, N200, and P300 components can be observed. On the other hand, statistical results indicate that the amplitudes of P200 induced at the PF2 lead, N200 induced at the F3 lead, and P300 induced at the C4 lead are significantly lower in ASD children compared to TD children, reflecting abnormalities in the mid-to-late stages of tactile processing in ASD, which implies that ASD has difficulties in differentiating tactile levels and forming tactile decisions. Combined with **Figure 4A**, we can identify the corresponding brain regions related to these mid-to-late stage abnormalities in the EEG components of ASD. It is evident that the brain regions recruited by ASD and TD differ across these three stages. The activation pattern in TD (late-stage C4 activation) is similar to the response to tactile stimulation reported in normal adults by Zhang et al. (21). In contrast, even in the late stages of tactile stimulation, the activation regions in ASD remain in the postcentral gyrus, an area typically activated in the early stages of tactile stimulation in normal adults (21). The above results suggest that information processing in the ASD group is delayed compared to the TD group. This phenomenon of sluggish tactile processing has also been reported in the behavioral experiments conducted by Katie et al., who found through behavioral studies that tactile brain processing in adult ASD individuals was delayed by 60 ms compared to adult TD individuals, while multisensory integration was delayed by 180 ms (39).

Interestingly, the abnormally activated regions observed during the tactile discrimination process in ASD (differences in brain regions FP2 and C4 for the five levels of tactile stimuli) are consistent with those identified in MRI studies (38), specifically in the prefrontal and sensorimotorareas, as illustrated in **Figure 4B**.

To further explore information processing during tactile recognition, particularly the efficiency of dynamic information processing, as illustrated in **Figure 5**. we conducted a dynamic coefficient of variation analysis and performed pair-wise comparisons between the two groups (ASD *vs*. TD). We found that ASD children process tactile information throughout the entire time period; however, the coefficient of variation within a single time window is lower. This means that ASD children require more time but achieve lower efficiency to complete tactile cognitive tasks. Considering the entire brain spatial region, the coefficient of variation in the C4 of ASD children is lower than that of TD children over the entire time period, while it is higher in the FP2. This indicates that, in the context of low tactile processing efficiency in the C4, ASD patients exhibit compensatory tactile processing behavior in the FP2. This finding may reveal the defects in tactile recognition in ASD. Piccardi and colleagues (16)sought to identify physiological markers for ASD, exploring the use of tactile EEG as a potential biomarker. They emphasized the importance of early neural markers in predicting the later development of ASD, they innovatively proposed using tactile EEG as a physiological marker. However, their study did not conduct a comprehensive temporal analysis of tactile processing EEG, but instead relied on a single measure, the power spectral density at 300 ms. Additionally, their paradigm was not designed according to the most prominent tactile abnormalities in ASD (resolution issues), which might have resulted in fewer significant brain regions being identified.

In summary, results showed significantly reduced ERP amplitudes in ASD children at electrodes FP2, F3, and C4, indicating deficits in mid-to-late tactile processing stages. Brain topography revealed key group differences in regions such as the F8, FC5, FC6, T8, CP1, CP2, CP5, CP6, Pz, and Oz. CV analysis indicated prolonged but inefficient tactile processing in ASD, with compensatory activation in the FP2. These findings suggest that ASD children exhibit delayed tactile processing and inefficient discrimination of tactile stimuli. The identified EEG patterns and cortical activation differences provide a foundation for developing objective, early screening methods for ASD.

Traditional early screening methods for ASD (e.g., ABC, CARS, CHAT, ADOS, ESAT) primarily rely on parent-reported questionnaires (40), requiring approximately 30–60 minutes depending on questionnaire complexity and parental comprehension. In contrast, our paradigm, including preparation, is completed in approximately 15 minutes, improving the efficiency of early screening processes. Simultaneously, the integration of EEG enables the objective quantification of neural responses (41), providing a more precise and reliable approach to assessing brain activity. Wang and colleagues examined functional connectivity patterns in ASD using resting-state EEG. The study collected eyes-open resting-state EEG data from 72 children with ASD and 63 TD children and applied a data-driven clustering method to classify ASD into two subgroups: mild ASD (mASD) and severe ASD (sASD). The results revealed increased functional connectivity in the beta band for mASD and decreased connectivity in the alpha band for sASD compared to TD children, demonstrating that EEG can effectively distinguish between ASD and TD. However, the limitation of resting-state EEG lies in its focus on frequency characteristics, as it cannot capture temporal or spatiotemporal features, restricting its ability to fully explore dynamic neural activity in ASD. Marsicano et al. (42) utilized a visuo-spatial attentional task combined with EEG to investigate the dynamics of visual attention in individuals with ASD. The study included 19 children with ASD (mean age 11.21 years) and 20 TD children (mean age 11.25 years). Participants responded to visual targets following “zoom-in” or “zoom-out” cues. The results demonstrated prolonged neural encoding of visual cues in the ASD group, persisting even after target onset, whereas in the TD group, cue-related activity rapidly diminished after target appearance. This study also confirmed delays in sensory processing in ASD, similar to the tactile processing delays observed in our study. However, the visual task paradigm relied on participants making explicit judgments about visual stimuli and required significant cognitive engagement, limiting its applicability to younger children, particularly those under 3 years old, thereby reducing its potential utility for early screening applications. Tactile EEG paradigms demonstrate high efficiency, low demands on participants, and the ability to analyze multidimensional EEG information, highlighting their potential for early screening applications. Tactile electrical stimulation paradigms also present the challenge of potentially causing discomfort. In the future, more comfortable wearable flexible tactile systems will be designed to enhance user comfort.

This study demonstrated good test-retest reliability of EEG features during tactile tasks through ICC analysis. Specifically, the pressure-level CV values showed ICC=0.779 (95%CI:0.712-0.832) in the ASD group and ICC=0.729 (95%CI:0.653-0.792) in the TD group, which is largely consistent with the ICC range (0.68-0.81) reported by Zhang et al. (43) for tactile tasks. Using these stable features, we achieved a classification accuracy of 0.852 (95%CI:0.812-0.887) on the independent test set. Compared to recent similar studies: this result is slightly lower than the 0.89 accuracy obtained by Wang et al. (44) using multimodal data, but outperforms the 0.79 accuracy reported by Chen et al. (45) using resting-state EEG alone. Notably, the ERP component at pressure level 4 (P200 wave at Fz electrode) demonstrated both high reliability (ICC=0.751) and strong discriminative power (accuracy=0.852). This finding provides a biomarker with both stability and specificity for objective ASD diagnosis.

In future research, we will consider incorporating tactile discrimination tasks that reflect specific tactile behaviors in ASD, such as their aversion to dynamic touch and preference for static heavy pressing. However, it is important to acknowledge several limitations of this study. Firstly, despite extensive literature highlighting the gender disparity in autism prevalence, the participants in this study exhibit a gender imbalance. To address this, future research will recruit gender-matched subjects to evaluate the potential impact of gender on the findings. Additionally, future studies will explore the coupling and transmission of information between different brain regions, employing methods such as dynamic brain network analyses. IQ and language skills of participants were not assessed due to the age range of our sample, which included children as young as 1 year and 2 months, making formal IQ assessments infeasible. This lack of data may limit the interpretation of the relationship between cognitive abilities and tactile processing, To address this limitation, future studies will incorporate standardized IQ and language skill assessments, such as the Mullen Scales of Early Learning (MSEL) or the Peabody Picture Vocabulary Test (PPVT), depending on the age and cognitive abilities of the participants. Integrating these measures will allow for a more comprehensive analysis of how cognitive and linguistic factors may influence tactile processing in ASD. The statistical approach employed for analyzing questionnaire data may require further scrutiny. In future investigations, we intend to incorporate qualitative analytic techniques—such as thematic analysis—and to recruit larger sample sizes, thereby enhancing the robustness and generalizability of our findings. Furthermore, correlating these cognitive and language assessments with EEG-based classification results could provide additional insights into the neural mechanisms underlying tactile processing and its relationship with broader developmental profiles in ASD. While our pressure levels were initially determined from healthy adult thresholds for experimental consistency, Future studies should incorporate such pediatric-specific calibration, particularly when comparing absolute sensitivity thresholds between groups. Designing experimental paradigms targeting these specific tactile behaviors will help more accurately identify significantly abnormal brain regions and provide a more comprehensive understanding of tactile processing in ASD. Once the mechanisms underlying tactile processing abnormalities in ASD are fully understood, future research will focus on recruiting a large number of participants to build an EEG feature database for tactile processing. Using artificial intelligence algorithms, we will classify the EEG features of ASD and TD individuals, aiming to develop a binary classification method for early screening, which could be applied to intelligent diagnostic tools for clinical early screening.

## 5 Conclusions

This study employed EEG-based analysis to investigate the neural mechanisms underlying tactile processing differences between children with ASD and TD children. The results revealed distinct neurophysiological patterns in ASD, characterized by significantly reduced amplitudes of mid-to-late ERP components (P200, N200, and P300) at key electrodes (FP2, F3, and C4), indicating impaired tactile discrimination and decision-making processes. Brain topography further highlighted group differences, with ASD children exhibiting atypical activation in regions such as F8, FC5, FC6, T8, CP1, CP2, CP5, CP6, Pz, and Oz, suggesting compensatory neural recruitment despite overall inefficiency in tactile processing. CV analysis demonstrated prolonged but less efficient tactile information processing in ASD, with compensatory activity observed in the FP2. These findings underscore the potential of EEG-derived biomarkers, such as ERP components and CV values, for objective early screening of ASD. The study also validated the reliability of these EEG features, with machine learning models achieving high classification accuracy, supporting their utility in clinical applications. In summary, this research provides novel insights into the neural basis of tactile processing abnormalities in ASD and lays the groundwork for developing non-invasive, objective diagnostic tools. Future studies should expand sample sizes, incorporate longitudinal designs, and explore multimodal approaches to further refine these biomarkers and enhance their clinical applicability.

## Data Availability

The raw data supporting the conclusions of this article will be made available by the authors, without undue reservation.
