# Supplementary figures and images for: Altered tactile abnormalities in children with ASD during tactile processing and recognition revealed by dynamic EEG features
Source: Front Psychiatry. 2025 Sep 16;16:1611438. doi: 10.3389/fpsyt.2025.1611438 (PMC12481717; doi:10.3389/fpsyt.2025.1611438)

## 1 Supplementary Material

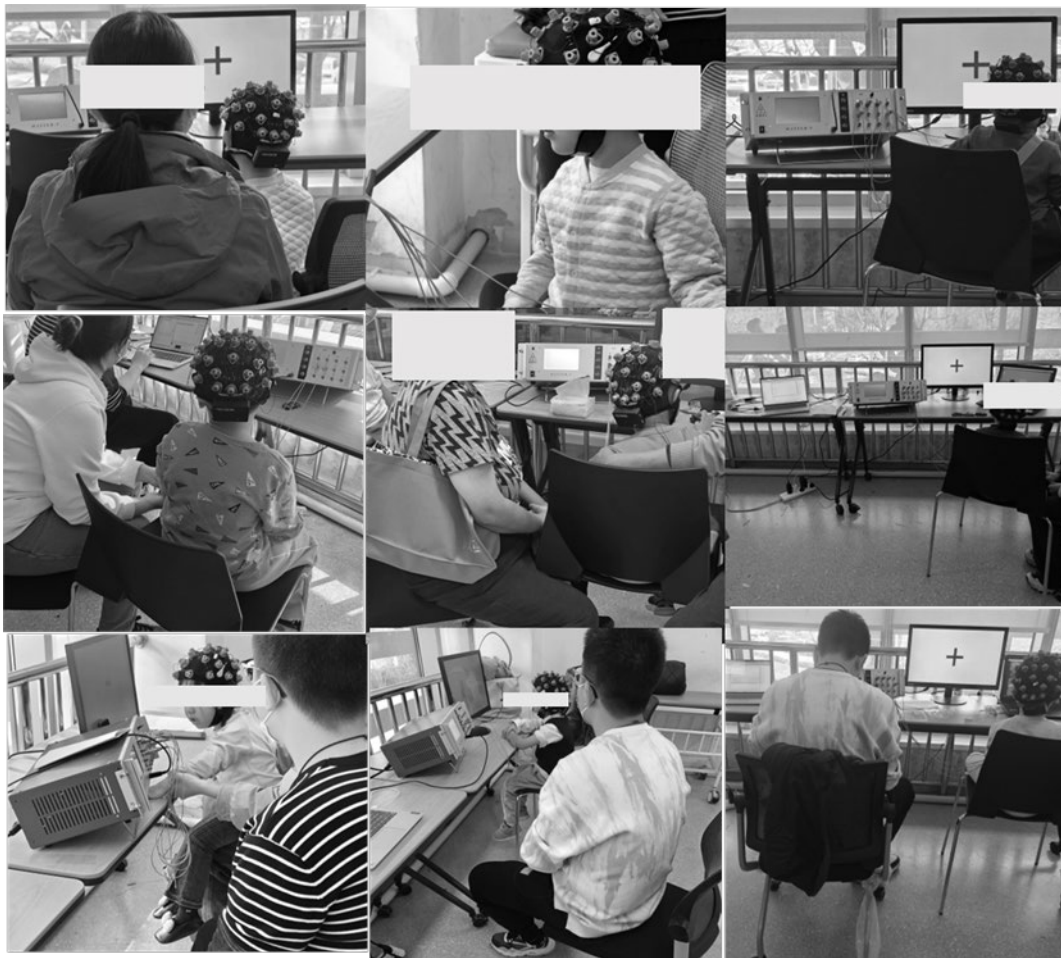

Supplementary Figure 1. Experimental Scenarios

Supplement: Supplementary file 1 [file Image1.pdf]
